# Investigation on heroin and cocaine quality in Luxembourg

**DOI:** 10.1186/s12954-021-00544-x

**Published:** 2021-09-16

**Authors:** Adèle Bourmaud, Georges Dahm, François Meys, Nicolas Gengler, Alain Origer, Serge Schneider

**Affiliations:** 1 Laboratoire national de santé, Service de toxicologie analytique – chimie pharmaceutique, 1, rue Louis Rech, 3555 Dudelange, Luxembourg; 2 Direction de la Santé, Ministère de la Santé, Allée Marconi - Villa Louvigny, 2120 Luxembourg, Luxembourg

**Keywords:** Heroin, Cocaine, Quality, Drug consumption facilities, Seizure

## Abstract

**Background:**

Heroin and cocaine are among the most dangerous illicit drugs available and their presence on the market is increasing. These facts have led to the investigation of the quality of heroin and cocaine samples seized in Luxembourg by police and customs but also collected at the national supervised drug consumption facilities.

**Methods:**

Samples obtained from 2019 to 2020 were analyzed to determine their composition and content using GC–MS, HPLC-UV and LC-Q-ToF. The statistical evaluation of concentration changes depending on the source of collection is based on an ANOVA single factor test and a two-tailed *t* test.

**Results:**

Results showed important differences between seizure and collection sources. For both drugs, customs samples had significantly higher concentrations than police samples and the latter had significantly higher concentrations than samples from drug consumption facilities, whereas for heroin two cutting steps were identified, for cocaine samples only one appears to occur on the local market. Indeed, cocaine samples seized by police consisted of a mixture of low and high concentration samples.

**Conclusion:**

The results show that extensive adulteration with pharmacological active and inactive compounds takes place at local levels, which, however, are different for heroin and cocaine. This knowledge on variability of quality of drugs should be considered in the elaboration of drug and harm prevention strategies.

## Introduction

In Europe, cocaine and heroin are considered being among the most dangerous psychoactive drugs sold on the illegal market when considering overall harm, that is to say physical, psychological and social effects to users and to others [1]. Furthermore, both products raise major concerns as cocaine is the second most frequently used illicit drug and heroin the most commonly used opioid. In 2018, cocaine seizures have increased to reach a peak with more than 181 tonnes and seizure volumes of heroin have almost doubled with 9.7 tonnes compared to 5.2 tonnes in 2017 [2].

Cocaine is extracted from coca plant leaves. Extracts are most often cut with adulterants (i.e., pharmacologically active substances) and diluents (i.e., pharmacologically inactive substances) [3]. Adulterants such as phenacetin, levamisole and lidocaine together with diluents such as mono- or polysaccharides are typically used. On the European market, cocaine is almost exclusively sold in the form of hydrochloride salt. In Luxembourg, it is the most prevalent controlled substance illegally sold after cannabis. Cocaine lifetime and last 12-month prevalence are estimated at 2.5% and 0.4%, respectively, of the national population aged 15 to 64 years [4].

Heroin is an opium extract consisting of diacetylated morphine, commonly adulterated with paracetamol and caffeine. In Western Europe, it is predominantly sold in the base form as brown/grey powder [5]. Black tar or white powder heroines are extremely rare in Luxembourg and in Europe in general. Heroin is commonly consumed through smoking/inhaling (47%) or intravenous injection after dissolution in an aqueous solution in presence of an acid (34%) [2]. Heroin, lifetime and last 12-month prevalence rates, in Luxembourg, are estimated at 0.6% and 0.1%, respectively [4].

Collection of data on drug quality mainly relies on the analysis of illicit products seized by customs (CUS) and police (POL). The determination of composition and quality of illegal heroin and cocaine is routinely performed by the National Laboratory of Health. Toxicological evidences are used for law enforcement, for criminal justice ruling but also for the drafting of national drug policies, intervention strategies and awareness campaigns. Thus, the national ministry of health is collecting data notably in the framework of its early warning system on controlled drugs and emerging drug use patterns. This enables decision making and actions to be implemented in case of new or unforeseen developments such as the detection of new or unsuspected adulterants or new psychoactive substances with potentially increased risks for health.

Several projects have been implemented nationally to increase data collection on the use of heroin and cocaine. These include a collaboration with national drug consumption rooms (DCR) to collect samples from their clients (± 200 per day). In return for providing a small amount of their substance, drug consumers obtain feedback on its quality.

In this paper, an investigation on heroin and cocaine quality, according to the source of sample collection is presented. The study includes samples from 2019 to 2020 collected by customs and police or obtained from DCR clients during the same period.

The goal was to assess drug quality on the local markets, to investigate quality differences according to various sample collection sources and thus, to provide reliable information for political decision makers and evidence-based drug policies.

## Material and methods

### Drug samples’ collection

Seized drug samples were received from customs and police for routine screening. Qualitative analyses were realized using GC–MS technique. X-ray fluorescence analyses and quantification of targeted drugs and adulterants by HPLC-UV were performed on a limited number of samples, representative of the seizure, as suggested by UNODC [6] and SWGDRUG [7]. If the number of identical samples (*n*) in a seizure was < 10 all of them were analyzed. If n was between 11 and 100 in a seizure, only 10 samples were analyzed. In the great majority of cases *n* was < 10, no seizures with more than 30 samples have been collected. Drug samples from heroin and cocaine users at DCRs were collected by social health care workers. These collected samples were analyzed using LC-Q-ToF to determine drug composition and content.

### Chemicals and reagents

Heroin (diacetylmorphine), paracetamol (acetaminophen), caffeine, cocaine, phenacetin, levamisole hydrochloride, lidocaine, all at 1 mg/mL were obtained from either Cerilliant (Diegem, Belgium), LGC Standards (Luckenwalde, Germany or Middlesex, UK) or Lipomed (Arlesheim, Switzerland). HPLC water, acetonitrile and methanol for HPLC-UV analyses were purchased from Biosolve (Dieuze, France). For LC-Q-ToF analyses, UPLC water with 0.1% formic acid (solvent A), UPLC acetonitrile with 0.1% formic acid and UPLC methanol with 0.1% formic acid were purchased from Fisher Chemical (Merelbeke, Belgium).

Acetic anhydride was obtained from VWR (Briare, France). Pyridine was bought from Sigma-Aldrich (Diegem, Belgium). Potassium dihydrogen phosphate and ethyl acetate were provided by Chem-Lab (Zedelgem, Belgium).

HPLC eluents for heroin quantification by HPLC-UV consisted of aqueous buffer adjusted to pH 2.18 using 20 mM potassium dihydrogen phosphate (solvent A) and methanol (solvent B). A 9/1 (*v*/*v*) mixture of A and B (solvent C) was used to dilute samples prior to injection. HPLC eluents for cocaine quantification by HPLC-UV consisted of aqueous buffer adjusted to pH 2.18 using 20 mM potassium dihydrogen phosphate (solvent A) and acetonitrile (solvent C). A 92/8 (*v*/*v*) mixture of A and B (solvent D) was used to dissolve and dilute samples prior to injection. LC eluents for LC-Q-ToF analyses were UPLC grade water with 0.1% formic acid (solvent E) and UPLC grade acetonitrile with 0.1% formic acid (solvent F).

### Qualitative analysis using GC–MS

About 10 mg of the sample was acetylated using 50 µL of pyridine plus 50 µL of acetic anhydride for 15 min at 80 °C. The mixture was evaporated to dryness using a gentle stream of nitrogen at 40 °C. The residue was dissolved in ethyl acetate/methanol (9/1, *v*/*v*). A volume of 1 µL was injected into the GC–MS system for analysis using splitless mode.

GC–MS analysis was done on a 6890 gas chromatograph (Agilent, Waldbronn, Germany) equipped with an HP-Ultra2 column (12 m × 0.33 µm) coupled to a 5977B mass selective detector. The front inlet temperature was 260 °C. Chromatographic separation was achieved by varying the oven temperature from 70 to 280 °C at a rate of 20 °C/min. The final temperature was held for 11.5 min. Total run time per sample was 24 min. Helium was used as carrier gas at a flow rate of 1.1 mL/min and the solvent delay was 4 min. Mass selective screening was from *m*/*z* 50 to 650 at a scan rate of 50 scans/s.

Identification was performed using retention time and against MS databases [8]. Identification was based on comparison with known retention time from reference compounds and simultaneous presence of at least 3 ions among which the molecular and base peak ions when compared to the MS database.

### FRX analysis

About 50 to 100 mg of each sample was analyzed on an ARL QUANT’X EDXRF spectrometer (Thermo Fisher Scientific, Madison, USA) to search for the presence of chlorine, as indicator for the presence of cocaine or heroin in the hydrochloride form.

### Heroin dosage with HPLC-UV

About 10 mg of each sample was weighted exactly and dissolved in 10 mL of methanol using an ultrasonic bath for 5 min. The solution was diluted per 100 in solvent C and 10 µL of this solution was injected into the HPLC-UV system.

LC separation was achieved on an Ultimate 3000 system (Thermo Fisher, Belgium) equipped with a Dionex Acclaim RSLC PolarAdvantage II column (100 mm × 2.1 mm × 2.2 µm). The oven temperature was set up at 50 °C. The separation was obtained using a gradient of B from 12 to 20% in 1.45 min, 28% at 2.69 min, 58% at 4 min with a flow rate of 0.7 mL/min. UV DAD was monitored from 208 to 375 nm, quantification wavelength was 208 nm. A 5-point calibration curve was established by injecting 1, 2.5, 5, 7.5 and 10 µL of a heroin, paracetamol and caffeine mixture containing 10 µg/mL of each compound.

Calibration acceptance criteria for each compound were: *r*^2^ ≥ 0.99, signal to noise ratio ≥ 10 and retention time for heroin at 2.81 min ± 0.5 min. Identification and quantification criteria for each compound were: peak match ≥ 950 compared to a reference spectrum, relative standard deviation of peak purity index ≤ 3%, signal to noise ratio ≥ 10.

### Cocaine dosage with HPLC-UV

Preparation of cocaine samples was the same as for heroin except that the final dilution occurred in solvent D.

LC separation was achieved on the same HPLC/UV system using the same column as for heroin. The oven temperature was set up at 50 °C. The separation was obtained using a gradient of C from 8 to 10% in 1.2 min, 23% at 1.5 min, 32% at 1.9 min, 50% at 3.9 min with a flow rate of 0.7 mL/min. UV DAD was monitored from 206 to 375 nm, quantification wavelength was 235 nm. A 5-point calibration curve was established by injecting 1, 2.5, 5, 7.5 and 10 µL of a mixture containing 10 µg/mL cocaine and adulterants such as levamisole, phenacetin, ketamine and others when considered necessary.

Calibration acceptance criteria and identification plus quantification criteria were identical to heroin samples’ analyses. For cocaine, retention time was 2.58 min ± 0.5 min.

### Qualitative and quantitative analyses using LC-Q-ToF

About 10 to 25 mg of each DCR sample was weighted exactly and dissolved in 1 mL of methanol using an ultrasonic bath for 5 min. The solution was diluted per 10,000 in a solvent mixture E/F (9/1, *v*/*v*)). Samples were analyzed on a G6550A ifunnel Q-ToF LC–MS system (Agilent, Waldbronn, Germany) equipped with a 1290 Infinity HPLC system. The separation was performed onto an Acquity UPLC BEH C18 column (130 Å, 1.7 µm, 1 mm × 50 mm) by using a gradient of solvents E and F. The LC and Q-ToF operating conditions have been described in detail in Dahm et al. [9].

### Statistical evaluation

The evaluation of concentration changes depending on the source of collection (i.e., police, customs and DCR) is based on two statistical approaches: an ANOVA single factor test (*α* = 0.05) and a two-tailed *t* test (*p* < 0.05). All concentrations presented in this article are expressed in percentage weight/weight.

## Results and discussion

### Drug collection

All samples were seized by customs and police or collected from DCR clients from January 2019 to December 2020.

The statistical evaluation on POL and CUS samples has been performed only on samples containing more than 1% heroin or cocaine. Concentrations below 1% were considered contaminations from other samples. All samples collected in DCR were included in the study regardless of the heroin or cocaine concentration as they were bought by the consumers as heroin or cocaine. Regardless of the source, speedballs (a mixture of cocaine and heroin) were not included in this study.

A total of 659 heroin and 1078 cocaine samples have been analyzed. A summary of sample origins, CUS, POL or DCR, is given in Table 1.

**Table 1:** Distribution of samples based on the source of collection

	Number of samples, (%)
*Heroin*	
CUS	71, (10.8%)
POL	438, (66.5%)
DCR	150, (22.8%)
*Cocaine*	
CUS	46, (4.3%)
POL	973, (90.3%)
DCR	59, (5.5%)

*CUS* customs, *POL* police, *DCR* drug consumption rooms

### Heroin quality

After identification of heroin by GC–MS, a FRX analysis was carried out to check for the presence of chlorine and other elements. Only the heroin base was detected. Nearly all heroin samples (99.5%) contained paracetamol (analgesic, antipyretic) and caffeine (stimulant). No other cutting agents were detected in any samples analyzed (e.g., fentanyl, fentanyl derivatives, griseofulvin, dextromethorphan, diphenhydramine, quinine, sugars). The addition of caffeine may be helpful for heroin to vaporize at lower temperature thus giving a faster rush when smoked and to counterbalance the sedative effects of heroin. Paracetamol may be added because of its analgesic properties and its bitter taste which can disguise poor quality heroin.

**Fig. 1:**
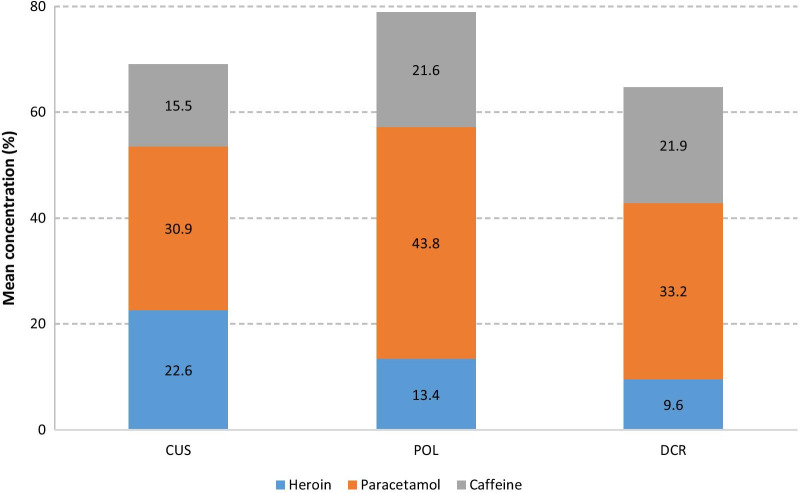
Composition of heroin samples from CUS, POL and DCR

The overall heroin concentrations varied from 0.1 to 55.6%. Mean, median and minimum heroin concentrations were highest in CUS samples, followed by POL and DCR samples. In DCR samples 2.7% were of very low quality (heroin < 1%) and 20.7% were of low quality (heroin 1–5%). In the POL seizures 10.3% of the samples had a heroin concentration < 5% and in the CUS seizures no sample contained less than 5% heroin. The heroin, paracetamol and caffeine concentrations are presented in Fig. 1.

On average, heroin concentrations in CUS samples were 168% and 235% higher than in POL and DCR samples, respectively. The significant difference was confirmed by an ANOVA single factor test (*α* = 0.05) and by a two-tailed *t* test (*p* < 0.05 between pairs).

Interestingly, the combined mean heroin/paracetamol/caffeine concentrations increased from CUS (69.0%) to POL (78.8%) and decreased from POL to DCR (64.7%) (Fig. 1). These findings suggest that first an adulteration step of the CUS samples using paracetamol and caffeine is performed followed by a cutting step with non-pharmacological compounds or the easily available caffeine by small-time dealers or end-users at DCR. Indeed, paracetamol and caffeine concentrations changed very similarly (factor about 1.4) when passing from CUS to POL accounting for the overall increase in the total heroin/paracetamol/caffeine concentration. From POL to DCR samples, the overall heroin/paracetamol/caffeine lowered by 14%, but relative caffeine concentrations were (almost) constant. This result is in accordance with reports of addition of caffeine, sand, or starch but not paracetamol at the DCR for cutting of heroin samples [10].

**Fig. 2:**
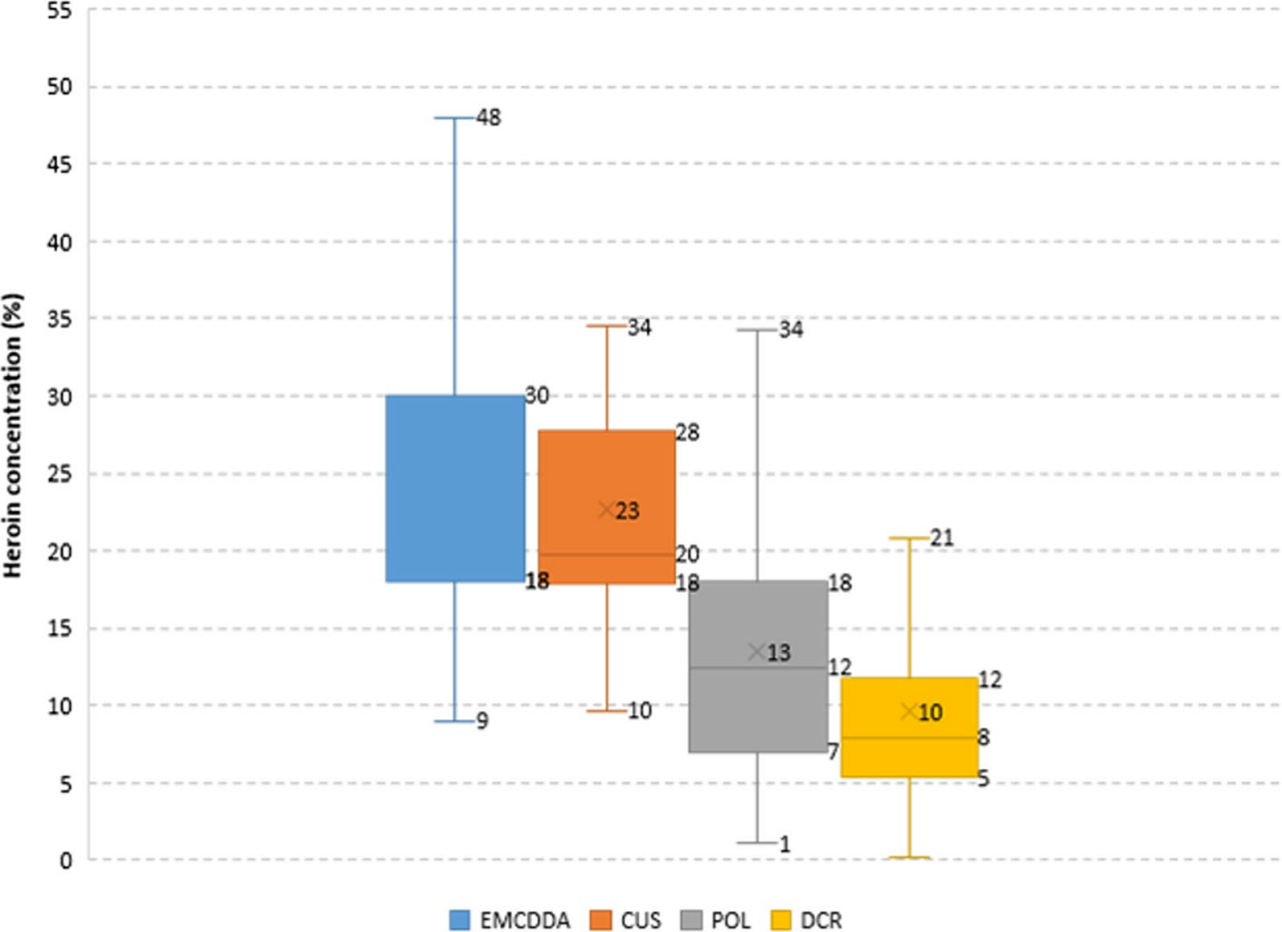
Distribution of heroin concentrations in CUS, POL, DCR samples and EMCDDA data

Only heroin samples seized by CUS showed similar concentrations than the ones reported for the European market by the European Monitoring Centre for Drugs and Drug Addiction (EMCDDA) for 2018 [2] (lower quartile Q1 18%, upper quartile Q3 30%, compared to 17.9% and 27.7% for the Luxembourgish CUS samples). As described above, POL and DCR were of lower quality, with Q1 7.0%, Q3 18.0% and Q1 5.3% Q3 11.7%, respectively (Fig. 2).

### Cocaine quality

As for heroin, a GC–MS analysis was performed to identify psychoactive or pharmacologically active drugs, adulterants and diluents. A FRX analysis showed that only cocaine chlorhydrate was available on the local market.

Main adulterants identified were levamisole (present in 54.2% of samples, anthelmintic allowed for veterinary use only), phenacetin (35.8%, antalgic, withdrawn from the European pharmaceutical market in the 1980s) and caffeine (10.5%, stimulant). Minor adulterants (< 2% samples) detected were lidocaine, ketamine, hydroxyzine. As only few CUS and DCR samples contained adulterants above the lower limit of quantification no statistical interpretation on adulterants was carried out.

There is much speculation about the rational of adding prohibited and withdrawn pharmacologically active substances to cocaine and no undisputed explanation has emerged so far. Possibly, lidocaine is added because some of its effects resemble those of cocaine (i.e., tachycardia, local anesthesia) and caffeine may be added, because it is a stimulant, thus enhancing the cocaine effects [11]. Diluents identified were carbohydrates such as glucose, sucrose and lactose. No quantification of the diluents has been performed.

**Fig. 3:**
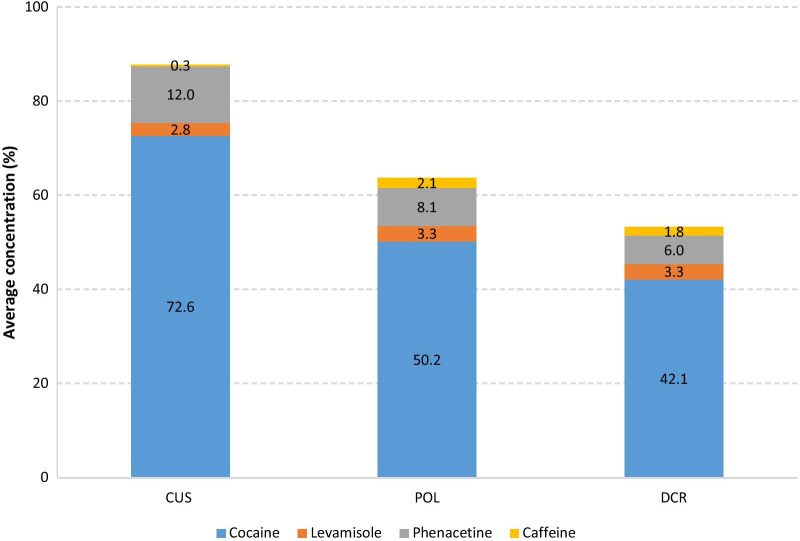
Composition of cocaine samples from CUS, POL and DCR

Cocaine samples are more heterogeneous than heroin samples. The concentration range varied from 3.4 to 100% cocaine chlorhydrate. Only one sample from POL and one sample from DCR were found to be of low quality (< 5%). A summary of the content of cocaine and its major adulterants is presented in Fig. 3.

Samples originating from CUS were in the concentration range 40.5 to 96.8% with a mean concentration of 72.6%. Samples originating from POL and DCR had mean cocaine concentrations of 50.2% and 42.1%, respectively. As for heroin samples, a significant decrease in cocaine content was found from CUS > POL > DCR, confirmed by an ANOVA single factor test (*α* = 0.05) and a two-tailed *t* test (*p* < 0.05). Thus, on average, cocaine concentrations in CUS samples were 143% and 171% higher than in POL and DCR, respectively.

**Fig. 4:**
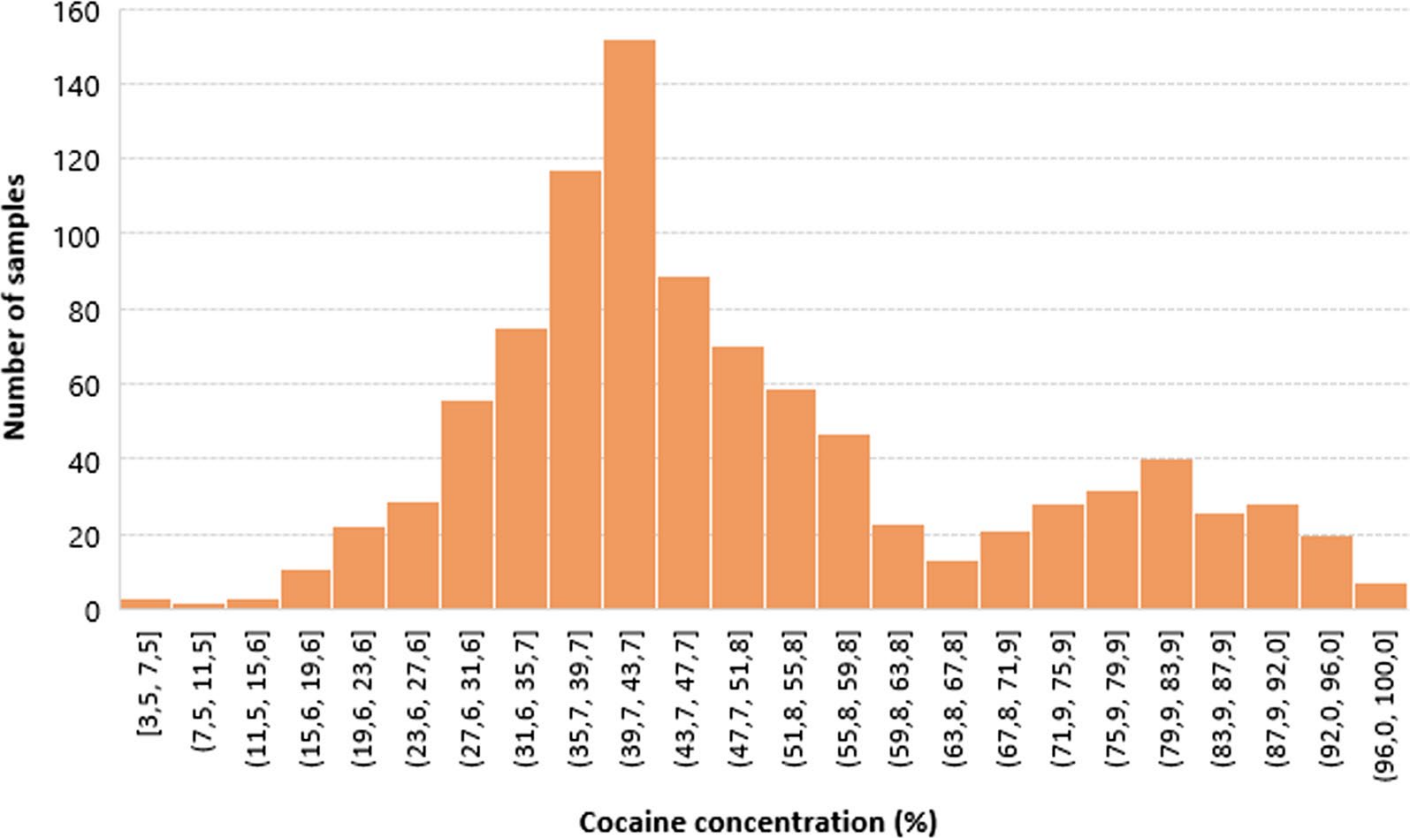
Distribution of cocaine purity in police samples

The combined cocaine/levamisole/phenacetin/caffeine concentrations decreased from CUS to POL to DCR samples as depicted in Fig. 3. However, contrary to heroin, this decline in quality and purity was not counterbalanced by an increase in adulterant concentrations. A closer look at the POL seizures also revealed that only two cocaine batches may be distinguished on the local market (Fig. 4): a first, of low concentration (about 40% cocaine) is similar to the DCR cocaine concentration (mean 42.1%) and may be corresponding to the products sold by small-level dealers and consumed by end-users. A second high concentrated population with a mean of about 80% is similar to the cocaine concentration found in CUS samples (72.6%) and may be corresponding to the samples used by higher-level dealers and end-consumers.

Unlike the heroin market where two cutting levels were observed, the cocaine market seems to be operating on a single cutting step: uncut samples found at CUS and POL or samples cut by a factor of ± 2, found at the POL and DCR. This is in concordance with two major consumer groups: ‘*high level*’ users (e.g., population with higher socio-economic background) using high-quality cocaine and socially marginalized groups (i.e., heroin addicts) consuming more cut, low-quality cocaine. To our knowledge, this has not been reported before and deserves further investigation at the European level.

Analysis of adulterants detected is difficult, because many are near or below LOD/LLOQ values. Thus, a significant qualitative difference could only be detected for caffeine. It was present in 6.5% of CUS and 6.3% in POL samples but 83.1% of the DCR samples. Overall, a cutting of CUS samples using caffeine and/or carbohydrates is the most plausible explanation for the significant decrease in cocaine concentrations in DCR samples.

**Fig. 5:**
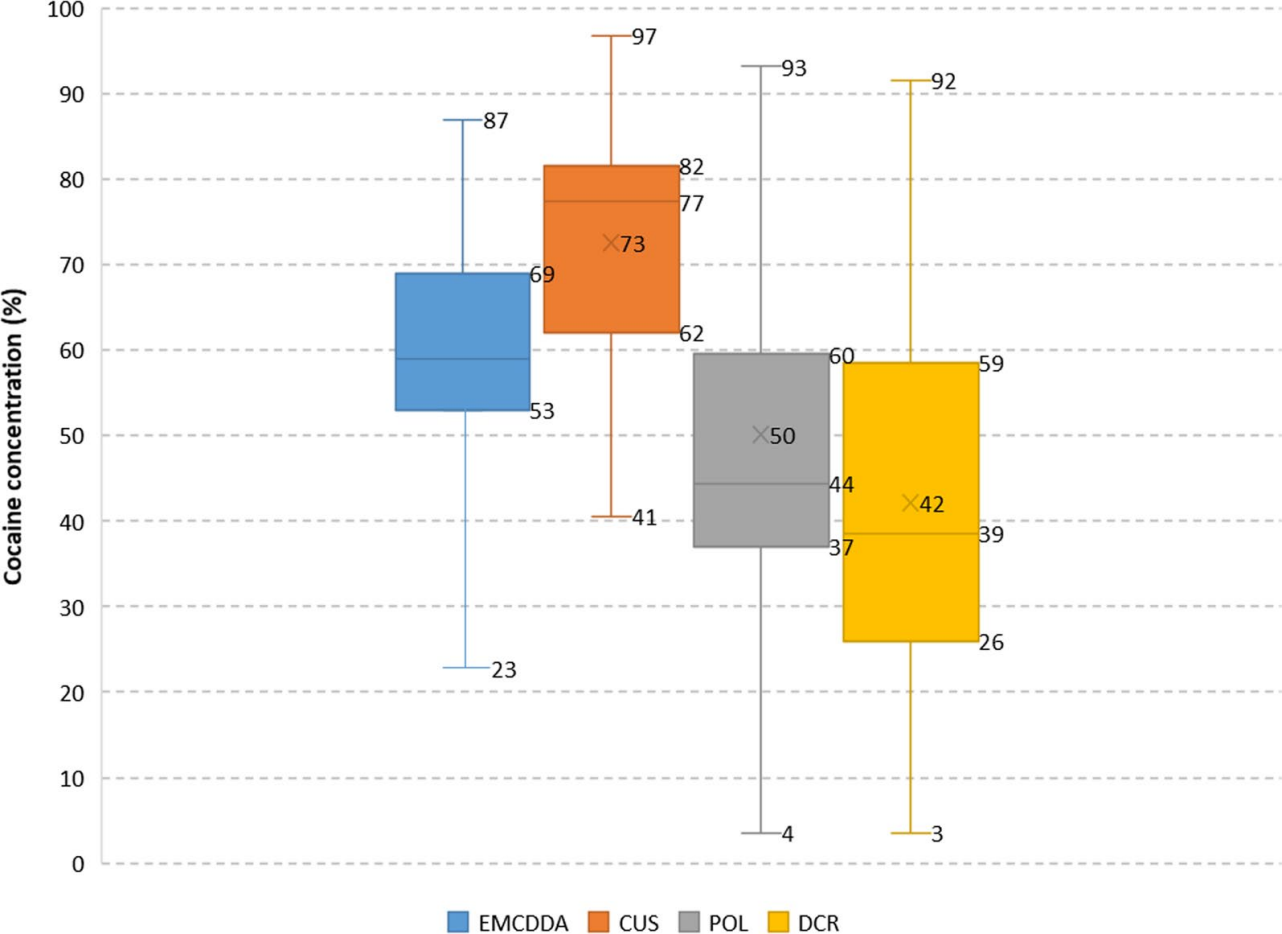
Concentration of cocaine in CUS, POL and DCR samples compared to EMCDDA data

When comparing cocaine samples from CUS, POL and DCR to EMCDDA values [2], as expected, the latter are in between the CUS and DRC values (Fig. 5) (EU lower quartile Q1 53%, upper quartile Q3 69%, compared to 26% and 59% for the DCR samples and 62% and 82% for the CUS samples).

## Conclusion

Toxicological analysis of heroin and cocaine samples seized by customs and police or collected from end-users at drug consumption rooms was carried out in 2019 and 2020.

No NPS (e.g., stimulants, synthetic cannabinoids), fentanyl or fentanyl derivatives were detected in any of the samples. For both heroin and cocaine, however, a significant decrease in concentration was observed from CUS to POL and from POL to DCR. Heroin concentrations decreased from 22.6% for CUS to 13.4% for POL and 9.6% for DCR samples. An adulteration with paracetamol and caffeine probably takes place in a first step followed by cutting with caffeine and/or non-pharmacological compounds.

Cocaine concentration also decreased from 72.6% for CUS to 50.2% for POL and 42.1% for DCR samples. The digressive gradient in quality may be explained by addition of sugars and/or caffeine as they are cheap, legal and readily available. Unlike heroin, POL cocaine samples revealed two different quality batches, one with high levels of cocaine, similar to the CUS samples, and one with low-quality cocaine, similar to the one found in DCR samples.

Our results show that cutting of heroin and cocaine occurs at different levels on their way to end users; presumably by a two steps’ process for heroin and in a single step for cocaine. Knowledge and surveillance of the cutting processes, adulteration practices and variability of quality and purity of street drugs should be considered in the elaboration of drug and harm prevention and law enforcement strategies. Also, more international research is needed to further assess quality and adulteration gradients along the distribution chain in controlled substances available on illicit markets in order to enrich evidenced-based drug policies.

## Data Availability

Not applicable.

## Abbreviations

CUS: Customs
DCR: Drug consumption rooms
EMCDDA: European Monitoring Centre for Drugs and Drug Addiction
POL: Police
